# Individual and Combined Occurrence of Mycotoxins in Feed Ingredients and Complete Feeds in China

**DOI:** 10.3390/toxins10030113

**Published:** 2018-03-07

**Authors:** Rui Ma, Lei Zhang, Meng Liu, Yong-Teng Su, Wen-Mei Xie, Ni-Ya Zhang, Jie-Fan Dai, Yun Wang, Shahid Ali Rajput, De-Sheng Qi, Niel Alexander Karrow, Lv-Hui Sun

**Affiliations:** 1 Department of Animal Nutrition and Feed Science, College of Animal Science and Technology, Huazhong Agricultural University, Wuhan 430070, China; marui6@webmail.hzau.edu.cn (R.M.); zhanglei6@webmail.hzau.edu.cn (L.Z.); liumeng0821@webmail.hzau.edu.cn (M.L.); zhangniya@mail.hzau.edu.cn (N.-Y.Z.); dr.shahidali@hotmail.com (S.A.R.); qds@mail.hzau.edu.cn (D.-S.Q.); 2 The Cooperative Innovation Center for Sustainable Pig Production, Wuhan 430070, China; 3 Jiangsu Aomai Bio-Technology Co., Ltd., Nanjing 211226, China; ytsu@aomai-bio.com (Y.-T.S.); wmxie@aomai-bio.com (W.-M.X.); 4 Sichuan Green Food Development Center, Chengdu 610041, China; daijiefan@126.com; 5 Hubei Gosign Bio-Feed Co., Ltd., Wuhan 430040, China; mr.wangyun@163.com; 6 Department of Animal Biosciences, University of Guelph, Guelph, ON N1G2W1, Canada; nkarrow@uoguelph.ca

**Keywords:** aflatoxin B_1_, zearalenone, deoxynivalenol, feedstuffs, China

## Abstract

The objective of this study was to investigate the individual and combined contamination of aflatoxin B_1_ (AFB_1_), zearalenone (ZEN) and deoxynivalenol (DON) in feedstuffs from different Provinces of China between 2016 and 2017. A total of 1569 samples, including 742 feed ingredients and 827 complete pig feed samples, were collected from various regions of China for mycotoxins analysis. The results showed that individual occurrence rates of AFB_1_, ZEN, and DON were more than 83.3%, 88%, and 74.5%, respectively, in all the tested samples. DON was the most prevalent contaminant, followed by ZEN and AFB_1_, with the average concentrations ranging from 450.0–4381.5 μg/kg, 2.3–729.2 μg/kg, and 1.3–10.0 μg/kg, respectively. Notable, 38.2%, 10.8%, and 0.6% of complete pig feeds were contaminated with DON, ZEN, and AFB_1_ over China’s regulatory limits, respectively. Moreover, over 75.0% analyzed samples were co-contaminated with two or three mycotoxins. In conclusion, the current study revealed that the feedstuffs in China were severely contaminated with DON, followed by ZEN and AFB_1_ during the past two years. These findings highlight the importance of monitoring mycotoxins in livestock feed and implementing feed management and bioremediation strategies to reduce mycotoxin exposure.

## 1. Introduction

Mycotoxins are a large group of fungal secondary metabolites, which are toxic to both animals and humans and that are mainly produced by five genera: *Aspergillus*, *Fusarium*, *Penicillium*, *Claviceps* and *Alternaria* [1]. To date, approximately 400 mycotoxins have been identified [2]. Aflatoxin B_1_ (AFB_1_), zearalenone (ZEN) and deoxynivalenol (DON) are recognized as the major mycotoxins contaminants in agricultural products, including maize, wheat, barley, peas, peanuts, millet, oily feedstuffs, and forage, and their by-products [3,4]. As primarily produced by *Aspergillus*, AFB_1_ is the most toxic mycotoxin, possessing hepatotoxic, mutagenic, carcinogenic, and teratogenic properties in many species of animals; it is also has been classified as a Group І carcinogen [5,6,7]. Both ZEN and DON are mainly produced by *Fusarium*. ZEN is an estrogenic toxin that competes with 17 β-estradiol for estrogen receptor binding, which consequently leads to fertility and reproductive problems [8,9]. In contrast, DON can induce anorexia, vomiting, and impairs immune function in various livestock species that involves inhibiting DNA, RNA, and protein synthesis [10,11,12,13].

Owing to the negative effect of mycotoxins on the health and performance of livestock, many countries have established safety standards for these toxins in feed and feed ingredients. The European Commission for example, set maximum levels of AFB_1_, ZEN, and DON at 5–20 μg/kg, 250 μg/kg, and 900 μg/kg, respectively, in all feed ingredients and complete feed [14,15]. Recently, the Chinese government updated livestock safety standards for AFB_1_, ZEN and DON (Table 1), which are 10 to 20 μg/kg, 100 to 250 μg/kg, and 1000 to 5000 μg/kg, respectively, for complete feeds [16].

Today, climate change and global warming are increasing susceptibility of staple crops, corn, wheat and soybean to fungal colonization and mycotoxin contamination, particularlyAFB_1_, ZEN and DON [17,18]. Thus, it will be important to monitor mycotoxin levels in livestock feed ingredients well into the future to maintain animal health and ensure the safety of human food products. China’s agriculture sector is contamination of mycotoxin due to, there are several climatic regions across the country, such as Yangtze and the Yellow River basins that are warm and humid with plenty of rainfall, which is favorable for mold growth and mycotoxin production in cereals [19,20,21]. Investigation into the prevalence of mycotoxin contamination in staple crops in China is required to help prevent exposure of livestock to mycotoxins and to ensure food and feed safety. However, such information is still very limited in China, especially regarding the co-occurrence of mycotoxins in feed ingredients. Therefore, this study was conducted to determine the contamination of individual and combined mycotoxins (AFB_1_, ZEN and DON) in feed and feed ingredients collected from various regions of China. These results can serve as an important reference for feed manufacturers, livestock producers and Chinese regulatory authorities involved in feed and food safety.

## 2. Results

### 2.1. Concentrations of AFB_1_ in Feed Ingredients and Complete Feeds

The occurrence of AFB_1_ in feed ingredients and complete feeds are summarized in Table 2. A total of 1016 samples, including 522 feed ingredients and 494 complete feeds samples, were collected between 2016 and 2017 to measure the concentration of AFB_1_. AFB_1_ was detected in 76.9–100% of feed ingredients and complete feeds, with the mean values ranging from 1.3 to 10.0 μg/kg. The highest median level of AFB_1_ was 11.5 μg/kg in corn bran harvested in 2016 and followed by 10.0 μg/kg in domestic DDGS from 2017. The maximum contamination of AFB_1_ was 67.6 μg/kg in corn harvested in 2016, followed by 49.2 μg/kg in wheat flour from 2016, 36.4 μg/kg in pig complete feed (powder) from 2016, and 32.0 μg/kg in corn harvested during 2017. Only 10 samples, including 6 corn, 1 wheat flour and 3 complete pig feed (powder) from 2016, which account for 1.0% of all the analyzed feed ingredients and complete feeds, were contaminated with AFB_1_ at levels exceeding Chinese safety standard concentrations (Table 1).

### 2.2. Concentrations of ZEN in Feed Ingredients and Complete Feeds

A total of 1155 samples, including 596 feed ingredients and 559 complete feeds samples, were collected between 2016 and 2017 to measure the concentration of ZEN (Table 3). ZEN was detected in 88.0–100% of feed ingredients and complete feeds, with the average concentrations from 2.3 to 729.2 μg/kg. The highest median level of ZEN was 729.2 μg/kg found in corn gluten meal from 2017, followed by 302.9 μg/kg in corn bran from 2016, and 258.7 μg/kg in domestic DDGS from 2016. The maximum contamination of ZEN was 1363.2 μg/kg in corn germ meal made in 2016, followed by 1268.6 μg/kg in corn bran from 2016, 1195.9 μg/kg in wheat middlings from 2016, 1169.2 μg/kg in imported DDGS from 2016, and 1109.7 μg/kg in pig complete feed (powder) from 2016, respectively. A total of 37 samples, which account for 3.2% of all the analyzed feed ingredients and complete feeds, were contaminated with ZEN more than the 500 μg/kg during 2016–2017. Notably, a total of 60 complete pig feeds, which account for 10.7% of all the complete pig feeds, were contaminated with ZEN at levels exceeding the Chinese safety standard concentration of 250 μg/kg (Table 1). 

### 2.3. Concentrations of DON in Feed Ingredients and Complete Feeds

A total of 1271 samples, including 687 feed ingredients and 584 complete feeds samples, were collected between 2016 and 2017 to measure the concentration of DON (Table 4). DON was detected in 74.5–100% of feed ingredients and complete feeds, with the average concentrations ranging from 450.0 to 4381.5 μg/kg. The highest median level of DON was 4701.4 μg/kg in wheat flour from 2016, followed by 4004.3 μg/kg in wheat harvested during 2016, 3547.2 μg/kg in domestic DDGS from 2017 and 3045.2 μg/kg in corn bran from 2016. The maximum concentration of DON was 12,633.3 μg/kg in wheat middlings from 2016, followed by 11,028.9 μg/kg in barley harvested in 2016, 10,437.6 μg/kg in complete pig feed (powder) from 2017, and 9556.8 μg/kg in wheat flour from 2016, respectively. A total of the 18 samples, which account for 1.4% of all the analyzed feed ingredients and complete feeds, were contaminated with DON at levels exceeding the 5000 μg/kg during 2016–2017. Notably, a total of 223 complete pig feeds, which account for 38.2% of all the complete pig feeds samples, were contaminated with DON at levels exceeding the Chinese safety standard concentration of 1000 μg/kg (Table 1).

### 2.4. Co-Contamination of AFB_1_, ZEN and DON in Feed Ingredients and Complete Feeds

AFB_1_, ZEN and DON were present as co-contaminates in feed ingredients and complete feeds samples collected between 2016 and 2017 (Table 5). The co-occurrence of AFB_1_ and ZEN, AFB_1_ and DON, ZEN, and DON, as well as AFB_1_, ZEN and DON, in feed ingredients ranged from 76.9–100%, 76.9–100%, 75.0–100% and 76.9–100%, respectively. While, the co-occurrence of AFB_1_ and ZEN, AFB_1_ and DON, ZEN, and DON, as well as AFB_1_, ZEN and DON in complete pig feed ranged from 96.4–100%, 96.4–100%, 97.9–100% and 96.4–100%, respectively.

## 3. Discussion

The current study was conducted to determine the individual and combined occurrence of three of the most prevalent and toxic mycotoxins (AFB_1_, ZEN and DON) in feed ingredients and complete pig feeds from different regions of China from 2016 to 2017. Generally, all three mycotoxins showed a quite high prevalence in the analyzed samples during the past two years, ranging from 74.5% to 100% in total. The mean level of AFB_1_ (1.6–10.0 μg/kg) measured in the current study was relatively lower than previously reported levels (0.4–627 μg/kg) in China during the period of 2012–2015 [21,22], while the percentage of samples containing AFB_1_ (76.9–100%) was very high, and 1.0% of the 1016 samples exceeded China’s safety standards. Notably, the relatively lower mean level of AFB_1_ in the current study can explain that only 1.2% of the whole tested complete pig feeds were contaminated with AFB_1_ over 10 μg/kg, which was much lower than those of the previously reported 7.7–7.8% [21,22]. This discrepancy may be because the analyzed feedstuffs were randomly collected from various areas, and weather varies in these regions during the collection period. A recently summarized report showed that the range values of AFB_1_ in feed materials and feedstuffs were 8 μg/kg, 2–3 μg/kg, 0–3 μg/kg, 8–90 μg/kg, 1 μg/kg, and 42 μg/kg in North America, Central South America, Europe, Asia, Oceania, and Africa, respectively [23]. The range value of AFB_1_ in feedstuffs in the current study was 0–67.6 μg/kg, which is similar to the value from Asia, which was the highest compared with other areas [23]. Differences in the occurrence of AFB_1_ among various geographical areas may be due to the differences of the seasonal and local weather conditions during critical plant growing stages. In addition, since feedstuffs are major goods for import and export between countries, 2.1% feed ingredients samples (>20.0 μg/kg) and 14.6% complete pig feed samples (>5.0 μg/kg) exceeded the European Commission regulation (250 μg/kg) should not be ignored [21]. Because AFB_1_ is the most carcinogenic mycotoxin, it will be necessary to continue monitoring AFB_1_ levels in feed and feed ingredients well into the future.

The percentage and concentration of the analyzed *Fusarium* mycotoxin ZEN in the present study were quite high. The percentage and range of mean ZEN concentrations in feed stuffs were 88.0–100% and 2.3–729.2 μg/kg, respectively, which is similar with previous Chinese reports showing 50.0–100% and 0–630.2 μg/kg, respectively, in feedstuffs between 2012 and 2015 [21,22]. ZEN was mainly contaminated in corn gluten meal, corn germ meal, corn bran, and DDGS, barley, wheat middlings and rice bran, which is similar to previous reports that showed that ZEN primarily occurred in corn, wheat and barley and their by-products in China, South Korean, Europe, Middle East, and Africa [21,22,24,25,26]. Also, 10.7% of 559 complete pig feed samples exceeded the regulatory limits in China, which is relatively lower than a previous investigation conducted in China [21]. This discrepancy may also be attributed to the different sampling areas and different weather conditions during the collection periods. Notably, although no feed ingredients samples exceeded the European Commission regulation (2000–3000 μg/kg), similar regulatory limits in European and China for complete pig feeds indicated that exporting these feeds should be strictly monitored [26]. Notably, the incidence rate and the mean range of ZEN concentrations in the complete pig feeds in China were higher than those of in South Korean which were 95% and 31.7 μg/kg, respectively [26]. Meanwhile, the recently summarized report showed that the range values of ZEN in feed materials and feedstuffs were 217 μg/kg, 0–111 μg/kg, 3–37 μg/kg, 32–219 μg/kg, 50 μg/kg, and 25 μg/kg in North America, Central South America, Europe, Asia, Oceania, and Africa, respectively [23]. The maximum value of ZEN in feedstuffs in the current study is 1363.2 μg/kg, which is higher than the values in all the reported areas [23]. Differences in the occurrence of ZEN among various geographical areas can be a result of the differences in the seasonal and local weather conditions during critical plant growing stages.

The percentage and concentration of the analyzed *Fusarium* mycotoxin DON in the present study were also high. The percentage of samples and average concentration of DON were 74.5–100% and 450.0–4381.5 μg/kg, respectively, which are similar to previous reports of 50.0–100% and 364.5–3931.7 μg/kg, respectively, in feedstuffs collected in China during the period of 2012–2015 [21,22]. The results indicate that wheat, barley, wheat flour, wheat middlings, wheat brain, DDGS and corn bran were seriously contaminated with DON, and 5.6% of the 306 feed ingredients exceeded the 5000 μg/kg safety concentration set by China. Surprisingly, 38.2% of the 584 complete pig feed samples were contaminated with DON at concentrations that exceeded the 1000 μg/kg regulatory limit set by China; this degree of contamination was much higher than the previously reported percent ages (14.0–23.5%) [20,21,22], which could be due again to the different regions and their weather conditions. Only four feed ingredients (barley, wheat middlings and wheat flour) over the relatively loose regulatory limits in Europe (8000–12,000 μg/kg), similar safety standards in Europe and China for the complete pig feeds indicate that export these feeds should be rigorously supervised [21]. The recently summarized report showed that the range values of DON in feed material and feedstuffs were 1947 μg/kg, 51–237 μg/kg, 88–968 μg/kg, 61–691 μg/kg, 94 μg/kg, and 745 μg/kg in North America, Central South America, Europe, Asia, Oceania, and Africa, respectively [23]. The maximum value of DON in feedstuffs in the current study is 12,633.3 μg/kg, which is at least 6 times higher than the values in all the reported areas [23]. Differences in the occurrence of DON among various geographical areas may be due to the differences in the seasonal and local weather conditions during critical plant growing stages. Taken together, this study indicates that the investigated feed ingredients and complete pig feeds in China during the past 2 years were severely contaminated with *Fusarium* mycotoxins, especially DON; and the government, feed company and farmers need to be aware of this.

Co-occurring mycotoxins may exhibit addictive or synergetic toxic effects, and this has been well documented by many studies [4,27,28,29,30]. Unfortunately, co-occurrence of mycotoxins was quite common in the present study, with more than 75% of samples contaminated with two or three mycotoxins. Notably, the DDGS, corn bran, corn germ meal, corn gluten meal, wheat, and rice bran samples were 100% co-contaminated with AFB_1_, ZEN and DON in all the tested samples, and more than 96.4% complete pig feeds were co-contaminated with these three mycotoxins. These outcomes were consistent with previous investigations that revealed that mycotoxin co-occurrence is an extremely common problem in feed industry world widely [23,25,31,32,33,34,35]. Because current safety regulations do not consider the toxic potential of co-occurring mycotoxins, the combined effects on animal and human health are probably under estimated; these combined toxic effects need to be investigated further and should be taken into consideration when new regulatory limits are set in the future.

It is also worth noting that the mean levels of the three analyzed mycotoxins in most of the feed ingredients and complete feeds were lower in 2017 compared to those of in 2016. The domestic DDGS had similar AFB_1_ concentrations compared to the imported DDGS in both 2016 and 2017, lower mean ZEN concentrations than imported DDGS in 2017, and much higher DON concentrations than the imported DDGS in both 2016 and 2017; the three analyzed mycotoxins concentrations appeared similar in both pellet and powder pig complete feeds.

## 4. Conclusions

In summary, the current study showed that AFB_1_, ZEN and DON were highly prevalent in all the analyzed feedstuffs from various regions of China between January 2016 and December 2017. DON exhibited the most serious contamination in feedstuffs, followed by ZEN and AFB_1_. Moreover, the co-occurrence of AFB_1_, ZEN and DON were extremely common in the tested feedstuffs. Particularly, the percentage of these co-occurring mycotoxins was 100% in DDGS, corn bran, corn germ meal, corn gluten meal, wheat, and rice bran samples, and over 96.4% in complete pig feeds. Overall, these findings warn us that (1) mycotoxin contamination in feedstuffs should be routinely monitored; (2) appropriate detoxification strategies for mycotoxins should be used in the feed industry; and (3) new regulatory limits for mycotoxins need to consider their co-occurrence in feedstuffs.

## 5. Materials and Methods 

### 5.1. Samples Collection and Preparation

A total of 1569 samples were collected at livestock farms, or feed companies from various locations in China between January 2016 and December 2017. There were a 742 feed ingredient samples including 287 corn, 86 domestic DDGS, 88 imported DDGS, 8 corn bran, 17 corn germ meal, 6 corn gluten meal, 13 wheat, 15 barley, 76 wheat bran, 40 wheat middlings, 22 wheat flour, 24 broken rice, 20 rice bran and 40 soybean meal, as well as 827 complete pig feed samples, including 461 pelleted and 366 powdered forms. These samples were mainly collected from Guangxi, Guangdong, Shandong, Jiangsu, Shanghai, Zhejiang, Fujian, Jiangxi, Hunan, Hubei, Anhui, Henan, Sichuan, Hebei, Shanxi, Ningxia, Gansu, Heilongjiang, Jilin, Liaoning, and Inner Mongolia provinces. Most of the feed and feed ingredient samples were analyzed for AFB_1_, ZEN and DON, while few of them have been analyzed for only 1 or 2 mycotoxins due to the lack of available quantity. All the samples were stored in bags at −20 °C until analysis. All the samples were ground to a fine powder according to the method described by the European Commission Regulation EC 152/2009 for the analysis [36]. 

### 5.2. Extraction of Myctoxins from Samples

Extractions of mycotoxins from samples were prepared as described in previous reports [21,22]. Briefly, 25 g of the ground samples were mixed with 100 mL of methanol:water (80:20, *v*/*v*) for AFB_1_ measurement, acetonitrile:water solution (84:16, *v*/*v*) for ZEN measurement or methanol:water (60:40, *v*/*v*) for DON measurement. The samples were blended at high speed for 3 min, and then filtered through Mycosep^®^ #226 (Romer Labs. Inc., Singapore). The solvent extracts were diluted with phosphate-buffered saline solution (PBS, pH 7.4), then filtered through immunoaffinity columns; ZearaStar (Romer Labs, Tulln, Austria) for ZEN, AokinImmunoClean CF AFLA and CF DON (Aokin AG, Berlin, Germany) for AFB_1_ and DON, respectively. After column washing with PBS and a methanol-water solution, the mycotoxins were eluted from the columns with methanol, and mycotoxins concentrated to dryness under anitrogenair steam. The mycotoxin residues were then immediately re-dissolved in a mobile phase described below, filtered through a Millex PTFE 0.22 μm filter (Merck, Tianjin, China), and analyzed by high-performance liquid chromatography (HPLC).

### 5.3. HPLC Analysis

The mycotoxins were quantified as previously described [21,22]. Briefly, AFB_1_ was analyzed with a reversed-phase HPLC/fluorescencedetection system (Agilent 1260, Agilent Technologies, Waldbronn, Germany) with a 360 nm excitation and 440 nm emission fluorescence detector. A C_18_ column (4.6 mm × 250 mm, 5 μm, Dikma, Shanghai, China) was employed with the limits of detection (LOD) and quantification (LOQ) set at 0.5 μg/kg and 1.5 μg/kg, respectively. The analysis was performed using a mobile phase of methanol:water:acetonitrile (30:60:10, *v*/*v*/*v*) at a flow rate of 1 mL/min. The temperature of the column was set at 30 °C. ZEN and DON were analyzed with a Shimadzu LC-20A binary gradient liquid chromatograph (Shimadzu Europa GmbH, Duisburg, Germany) equipped with a C_18_ (4.6 mm × 150 mm, 5 μm) reversed-phase column (ZORBAX Eclipse XDB-C18, Agilent Technologies, Waldbronn, Germany). ZEN analysis was conducted using a mobile phase of methanol:water:acetonitrile (8:46:46, *v*/*v*/*v*) at a flow rate of 1 mL/min and detected under 274 nm excitation and 440 nm emission wavelengths [37];the LOD and LOQ for ZEN were 10 μg/kg and 24 μg/kg, respectively. DON was analyzed using a mobile phase of methanol:water solution (20:80, *v*/*v*) at a flow rate of 0.8 mL/min under UV light at a wavelength of 218 nm [38] and the LOD and LOQ for DON were 100 μg/kg and 260 μg/kg, respectively. The validity of mycotoxin peaks in HPLC chromatograms have been shown in Figure S1. The blank samples are the solvents that were used to dissolve standard samples before HPLC detection. LOD and LOQ correspond to the analyte amount for which the signal-to-noise ratio is equal to 3 and 10 [39,40], respectively, with a minor adjustment according to our previous study [22].

### 5.4. Statistical Analysis

All the data were calculated by Microsoft Excel 2010 (Microsoft Corporation, Redmond, WA, USA) and expressed as percentages or means, median and maximum.

## Figures and Tables

**Table 1:** Chinese safety standards for AFB_1_, ZEN and DON in feedstuffs of China ^1^.

Feedstuff	Maximum Limit (μg/kg)
AFB_1_	
Corn by-products and peanut cake	50
Vegetable oil (except corn oil and peanut oil)	10
Corn oil and peanut oil	20
Other plant feed ingredients	30
Complete feeds for young pigs and poultry	10
Complete feeds for growing-boilers and meat-duck and laying-ducks	15
Other complete feeds	20
ZEN	
Corn and its by-products (except Corn bran and corn steep powder)	500
Corn bran and corn steep powder	1500
Other plant feed ingredients	1000
Complete feed for young pigs	150
Complete feed for young gilts	100
Other complete feeds for pigs	250
Other complete feeds	500
DON	
Plant feed ingredients	5000
Complete feeds for pigs	1000
Other complete feeds	5000

^1^ AFB_1_, aflatoxin B_1_; DON, deoxynivalenol; ZEN, zearalenone.

**Table 2:** AFB_1_ concentrations in feed ingredients and complete feeds ^1^.

Item	Year	No. of Samples	Positive Samples (μg/kg)				Numbers of Samples in the Range (μg/kg)				
			%	Mean	Median	Maximum	<0.5	0.5–10	10–30	30–50	50–100
Corn	2016	175	94.9	5.8	4.3	67.6	9	156	5	1	4
	2017	75	97.3	4.1	3.3	32.0	2	70	2	1	0
Domestic DDGS	2016	38	100	9.8	10.0	19.7	0	19	19	0	0
	2017	1	100	3.1	3.1	3.1	0	1	0	0	0
Imported DDGS	2016	27	100	7.3	8.4	15.0	0	23	4	0	0
	2017	3	100	4.9	4.8	5.6	0	3	0	0	0
Corn bran	2016	5	100	10.0	11.5	13.5	0	2	3	0	0
	2017	3	100	4.6	4.2	5.8	0	3	0	0	0
Corn germ meal	2016	9	100	8.1	8.3	13.5	0	8	1	0	0
	2017	5	100	5.4	4.2	10.9	0	4	1	0	0
Corn gluten meal	2016	4	100	8.0	8.0	10.3	0	3	1	0	0
	2017	2	100	6.3	6.3	7.1	0	2	0	0	0
Wheat	2016	14	100	2.3	2.1	4.9	0	14	0	0	0
Barley	2016	6	83.3	1.6	1.7	2.8	1	5	0	0	0
Wheat bran	2016	45	100	2.5	2.4	6.4	0	45	0	0	0
	2017	16	100	2.7	2.8	3.8	0	16	0	0	0
Wheat middlings	2016	22	86.4	2.7	2.3	4.5	3	19	0	0	0
	2017	2	100	3.1	3.1	4.3	0	2	0	0	0
Wheat flour	2016	9	88.9	7.5	2.5	49.2	1	7	0	1	0
	2017	2	100	1.6	1.6	1.9	0	2	0	0	0
Broken rice	2016	13	76.9	3.6	2.1	16.7	3	9	1	0	0
Rice bran	2016	11	100	3.4	3.1	6.6	0	11	0	0	0
	2017	4	100	3.7	3.8	4.37	0	4	0	0	0
Soybean meal	2016	23	95.7	4.5	4.9	6.9	1	22	0	0	0
	2017	8	87.5	2.7	3.2	3.7	1	7	0	0	0
Complete pig feed (pellet)	2016	111	96.4	3.5	3.3	26.6	4	106	1	0	0
	2017	9	100	2.6	2.8	3.9	0	9	0	0	0
Complete pig feed (powder)	2016	155	100	4.0	3.7	36.4	0	151	3	1	0
	2017	219	97.7	3.6	3.5	25.7	5	213	1	0	0

^1^ Positive samples are defined as those with AFB_1_ ≥ 0.5 μg/kg (LOD). AFB_1_, aflatoxin B_1_; DDGS, dried distillers grains with soluble.

**Table 3:** ZEN concentrations in feed ingredients and complete feeds ^1^.

Item	Year	No. of Samples	Positive Samples (μg/kg)				Numbers of Samples in the Range (μg/kg)			
			%	Mean	Median	Maximum	<10	10–250	250–500	500–2000
Corn	2016	183	93.4	104.1	76.6	624.3	12	157	9	5
	2017	86	90.7	55.0	32.1	296.8	8	76	2	0
Domestic DDGS	2016	46	100	299.4	258.7	956.7	0	20	22	4
	2017	1	100	49.3	49.3	49.3	0	1	0	0
Imported DDGS	2016	33	100	274.8	212.7	1169.2	0	19	12	2
	2017	3	100	204.0	144.1	378.1	0	2	1	0
Corn bran	2016	6	100	432.1	302.9	1268.6	0	3	1	2
	2017	3	100	231.5	168.8	456.6	0	2	1	0
Corn germ meal	2016	10	100	316.5	188.7	1363.2	0	6	3	1
	2017	8	100	129.6	100.8	325.4	0	7	1	0
Corn gluten meal	2016	5	100	494.9	139.8	1095.1	0	3	0	2
	2017	2	100	729.2	729.2	1006.3	0	0	1	1
Wheat	2016	14	100	2.3	2.1	4.9	0	8	0	0
Barley	2016	14	100	154.4	148.4	393.8	0	12	2	0
Wheat bran	2016	50	88.0	94.0	81.8	439.3	6	41	3	0
	2017	20	95	89.3	65.8	304.7	1	18	1	0
Wheat middlings	2016	32	96.9	179.5	119.5	1195.9	1	24	6	1
	2017	5	100	81.4	57.9	180.8	0	5	0	0
Wheat flour	2016	8	90	111.7	99.5	330.9	1	6	1	0
	2017	2	50	37.2	37.2	79.2	1	1	0	0
Broken rice	2016	13	100	68.7	22.7	257.3	0	12	1	0
Rice bran	2016	13	100	282.3	149.5	879.8	0	9	1	3
	2017	4	100	169.1	169.9	280.0	0	3	1	0
Soybean meal	2016	27	96.3	76.9	69.2	202.4	1	26	0	0
	2017	8	100	38.8	32.9	56.9	0	9	0	0
Complete pig feed (pellet)	2016	123	100	210.7	129.5	916.5	0	87	23	13
	2017	9	100	55.7	42.5	138.5	0	9	0	0
Complete pig feed (powder)	2016	187	99.5	129.3	100.8	1109.7	1	170	14	2
	2017	240	99.6	65.1	43.9	597.8	1	231	7	1

^1^ Positive samples are defined as those with ZEN ≥ 10 μg/kg (LOD). DDGS, dried distillers grains with soluble; ZEN, zearalenone.

**Table 4:** DON concentrations in feed ingredients and complete feeds ^1^.

Item	Year	No. of Samples	Positive Samples (μg/kg)				Numbers of Samples in the Range (μg/kg)			
			%	Mean	Median	Maximum	<100	100–1000	1000–5000	>5000
Corn	2016	187	98.4	857.4	718.4	4590.8	3	130	54	0
	2017	97	97.9	750.3	639.6	2250.9	2	72	23	0
Domestic DDGS	2016	48	100	2599.7	2458.21	6044.7	0	2	45	1
	2017	2	100	3547.2	3547.2	5406.8	0	0	1	1
Imported DDGS	2016	34	100	1855.4	1717.1	4044.7	0	3	31	0
	2017	55	74.5	872.8	523.6	7297.8	14	16	24	1
Corn bran	2016	6	100	2943.2	3045.2	4710.7	0	1	5	0
	2017	3	100	1295.3	1086.4	1916.7	0	1	2	0
Corn germ meal	2016	10	100	1426.5	1229.2	2900.6	0	4	6	0
	2017	7	100	1206.9	1001.4	2374.6	0	3	4	0
Corn gluten meal	2016	4	100	1688.1	1665.2	2229.1	0	0	4	0
	2017	2	100	559.9	559.9	620.3	0	2	0	0
Wheat	2016	14	100	3613.8	4004.3	6595.6	0	2	10	2
Barley	2016	15	100	2635.8	492.6	11,028.9	0	9	3	3
Wheat bran	2016	53	100	2304.2	2405.6	6054.4	0	8	44	1
	2017	23	100	1394.6	1014.3	5642.1	0	9	13	1
Wheat middlings	2016	34	100	2961.2	2647.6	12,633.3	0	2	30	2
	2017	7	100	1543.0	1017.7	3363.0	0	3	4	0
Wheat flour	2016	9	100	4381.5	4701.4	9556.8	0	1	4	4
	2017	3	100	450.0	456.4	736.9	0	3	0	0
Broken rice	2016	13	100	1607.3	1038.7	4075.4	0	6	7	0
Rice bran	2016	16	100	1532.7	1505.1	3148.5	0	6	10	0
	2017	4	100	1271.6	1117.3	1900.0	0	1	3	0
Soybean meal	2016	29	96.6	451.6	377.9	1171.4	1	26	2	0
	2017	12	100	610.7	498.3	1478.5	0	11	1	0
Complete pig feed (pellet)	2016	128	99.2	1194.0	1089.6	4279.3	1	53	74	0
	2017	9	100	753.1	590.4	1690.9	0	7	2	0
Complete pig feed (powder)	2016	195	100	1018.1	936.8	3400.9	0	108	87	0
	2017	252	98	876.3	706.2	10,437.6	5	187	58	2

^1^ Positive samples are defined as those with DON ≥ 100 μg/kg (LOD). DDGS, dried distillers grains with soluble; DON, deoxynivalenol.

**Table 5:** Percentage of AFB_1_, ZEN and DON co-contaminants in feed ingredients and complete feeds ^1^.

Item	Year	AFB_1_ & ZEN (%)	AFB_1_ & DON (%)	ZEN & DON (%)	AFB_1_ & ZEN & DON (%)
Corn	2016	92.4	94.7	93.4	92.4
	2017	92.0	97.3	90.7	92.1
Domestic DDGS	2016	100	100	100	100
	2017	100	100	100	100
Imported DDGS	2016	100	100	100	100
	2017	100	100	100	100
Corn bran	2016	100	100	100	100
	2017	100	100	100	100
Corn germ meal	2016	100	100	100	100
	2017	100	100	100	100
Corn gluten meal	2016	100	100	100	100
	2017	100	100	100	100
Wheat	2016	100	100	100	100
Barley	2016	83.3	83.3	75.0	83.3
Wheat bran	2016	86.7	100	88.0	86.7
	2017	93.8	100	95.0	93.8
Wheat middlings	2016	86.4	86.4	96.9	86.4
	2017	100	100	100	100
Wheat flour	2016	87.5	87.5	87.5	87.5
	2017	100	100	100	100
Broken rice	2016	76.9	76.9	100	76.9
Rice bran	2016	100	100	100	100
	2017	100	100	100	100
Soybean meal	2016	91.3	95.7	96.3	91.3
	2017	87.5	87.5	100	87.5
Complete pig feed (pellet)	2016	96.4	96.4	99.2	96.4
	2017	100	100	100	100
Complete pig feed (powder)	2016	99.3	100	99.5	99.3
	2017	97.7	97.6	97.9	97.6

^1^ Co-occurrence of mycotoxins samples is defined as those simultaneously contain AFB_1_, ZEN, and(or) DON ≥ 0.5 μg/kg, 10 μg/kg, and 100 μg/kg (LOD), respectively. AFB_1_, aflatoxin B_1_; DON, deoxynivalenol; ZEN, zearalenone; AFB_1_ & ZEN, feedstuffs co-contaminated with AFB_1_ and ZEN; AFB_1_ & DON, feedstuffs co-contaminated with AFB_1_ and DON; ZEN&DON, feedstuffs co-contaminated with ZEN and DON; AFB_1_ & ZEN & DON, feedstuffs co-contaminated with AFB_1_, ZEN and DON; DDGS, dried distillers grains with soluble.

## Supplementary Materials

The following are available online at http://www.mdpi.com/2072-6651/10/3/113/s1, Figure S1: The HPLC chromatogram of AFB1 (A), ZEN (B), and DON (C).
