# Supplementary material for: Individual and Combined Occurrence of Mycotoxins in Feed Ingredients and Complete Feeds in China
Source: Toxins (Basel). 2018 Mar 7;10(3):113. doi: 10.3390/toxins10030113 (PMC5869401; doi:10.3390/toxins10030113)
Supplement: Supplementary file 1 [file toxins-10-00113-s001.docx]

Supplementary Materials: Individual and Combined Occurrence of Mycotoxins in Feed Ingredients and Complete Feeds in China

Rui Ma, Lei Zhang, Meng Liu, Yong-Teng Su, Wen-Mei Xie, Ni-Ya Zhang, Jie-Fan Dai, Yun Wang, Shahid Ali Rajput, De-Sheng Qi, Niel Alexander Karrow and Lv-Hui Sun


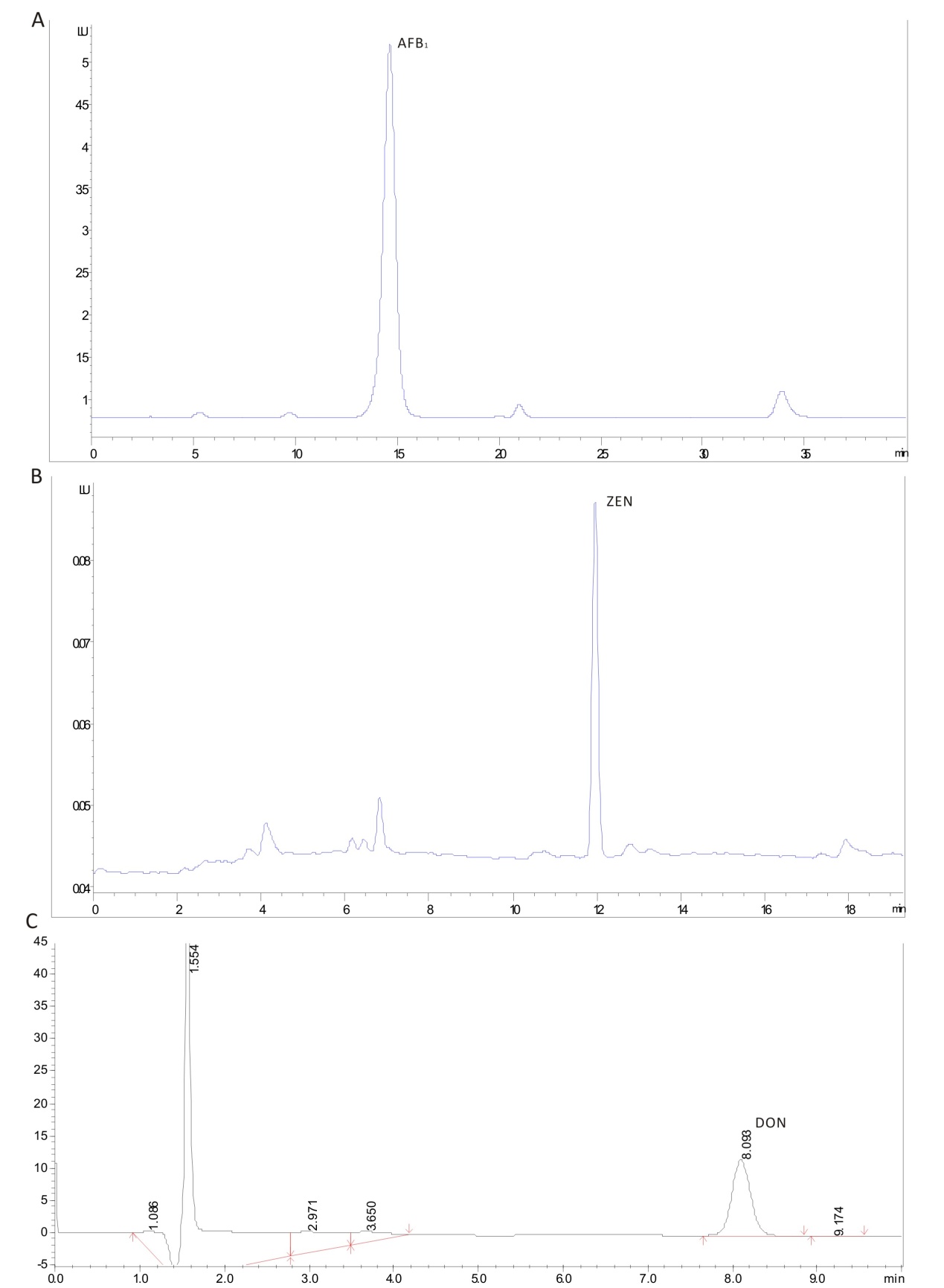


**Figure S1.** The HPLC chromatogram of AFB1 (A), ZEN (B), and DON (C).
